# Effects of intensive rehabilitation on functioning in patients with mild and moderate Charcot–Marie-Tooth disease: a real-practice retrospective study

**DOI:** 10.1007/s10072-023-06998-0

**Published:** 2023-08-08

**Authors:** Francesco Ferraro, Dario Calafiore, Claudio Curci, Francesco Fortunato, Irene Carantini, Filippo Genovese, Giuseppe Lucchini, Andrea Merlo, Antonio Ammendolia, Alessandro de Sire

**Affiliations:** 1 Physical Medicine and Rehabilitation Unit, Department of Neurosciences, ASST Carlo Poma, 46100 Mantova, Italy; 2 Institute of Neurology, Department of Medical and Surgical Sciences, University of Catanzaro “Magna Graecia”, 88100 Catanzaro, Italy; 3 ACMT-Rete per la malattia di Charcot-Marie-Tooth OdV Association, Rome, Italy; 4 Biostatistical Service, ASST Carlo Poma, 46100 Mantova, Italy; 5 LAM-Motion Analysis Laboratory, Neuromotor and Rehabilitation Department, San Sebastiano Hospital, Azienda USL-IRCCS di Reggio Emilia, Correggio, Reggio Emilia, Italy; 6 Physical Medicine and Rehabilitation Unit, Department of Medical and Surgical Sciences, University Hospital “Mater Domini”, University of Catanzaro Magna Graecia, Via Campanella, 115–88100 Catanzaro, Italy

**Keywords:** Charcot–Marie-Tooth disease, Physiotherapy, Rehabilitation, Pain, Balance, Neurological rehabilitation

## Abstract

Charcot–Marie-Tooth (CMT) disease is one of the most common inherited neuropathies and can lead to progressive muscular weakness, pes cavus, loss of deep tendon reflexes, distal sensory loss, and gait impairment. There are still no effective drugs or surgical therapies for CMT, and supportive treatment is limited to rehabilitative therapy and surgical treatment of skeletal deformities. Many rehabilitative therapeutic approaches have been proposed, but timing and cadence of rehabilitative intervention are not clearly defined, and long-term follow-up is lacking in literature. The aim of this real-practice retrospective study was to assess the effectiveness of an intensive neurorehabilitation protocol on muscle strength and functioning in CMT patients. We analyzed data of patients with diagnosis of mild to moderate CMT. The rehabilitation program lasted 2–4 h a day, 5 days a week, for 3 weeks and consisted of manual treatments, strengthening exercises, stretching, core stability, balance and resistance training, aerobic exercises, and tailored self-care training. Data were collected at baseline (T_0_), after treatment (T_1_), and at the 12-month mark (T_2_) in terms of the following outcome measures: muscle strength, pain, fatigue, cramps, balance, walking speed, and ability. We included 37 CMT patients with a median age of 50.72 ± 13.31 years, with different forms: demyelinating (*n* = 28), axonal (*n* = 8), and mixed (*n* = 1). After intensive rehabilitation treatment, all outcomes significantly improved. This improvement was lost at the 1-year mark. Taken together, these findings suggest that an intensive rehabilitation program improves short-term symptoms and functional outcomes in a cohort of inpatients affected by mild to moderate CMT.

## Introduction

Charcot–Marie-Tooth (CMT) neuropathy, also denoted as hereditary motor and sensory neuropathy (HMSN), represents the most frequent inherited neuromuscular disorder [1]. It is a genetically highly heterogeneous group with more than 80 genes identified when related neuropathies (hereditary sensory and autonomous neuropathy (HSAN), hereditary motor neuropathies (HMN)) are included [2]. Although CMT prevalence is estimated to be about 1:2500, the European prevalence rate is believed to be 10–28/100 000, but epidemiological studies are still scarce, and knowledge of CMT frequency in different parts of the world remains extremely limited [3, 4]. Moreover, the genetic heterogeneity manifests in different patterns of inheritance, such as autosomal dominant, autosomal recessive, and X-linked, as well as in distinct electrophysiological classes, such as axonal, demyelinating, and intermediate [1]. Two main subgroups can be defined based on electrophysiological and histopathological characteristics: the demyelinating form (CMT1), resulting from primary damage of myelinating Schwann cells (SCs), and the axonal form (CMT2), affecting the axons from motor (MNs) and/or sensory neurons (SNs). Patients affected with CMT1 have reduced nerve-conduction velocities (NCV, ≤38 m/s), whereas patients affected with CMT2 show slightly reduced to normal NCVs but reduced amplitudes (≥38 m/s) [5]. Clinically, CMT diseases are characterized by progressive muscular weakness starting at the distal extremities, pes cavus deformity, and loss of deep tendon reflexes, associated with mild to moderate distal sensory loss [6, 7]. CMT involves both the upper and lower limbs, causing hypotonia and hyposthenia of the foot and leg intrinsic muscles, and slowly progresses to the hands and forearms [8]. Due to the altered muscular balance and foot deformities, gait patterns have been described based upon stance and/or swing phase function or the ability to heel and toe walk, which reflect impairments related to strength [9, 10]. Patients commonly complain of walking difficulties, ankle twisting, tripping, postural in-stability, and frequent falls [7, 11]. Moreover, the disease includes also sensorial and respiratory alterations and other overlooked symptoms like cramps, fatigue, and pain that can significantly affect a patient’s quality of life [12–14]. Difficulties and energy expenditure when walking and performing ordinary tasks can further reduce mobility in people with CMT, thus leading to varying degrees of disability [15, 16].

To date, there are still no effective drugs or surgical therapy for CMT [16]. Supportive treatment is limited to rehabilitative therapy and surgical treatment of skeletal deformities and soft-tissue abnormalities, and rehabilitation might play a pivotal role in CMT management [17–22]. Many rehabilitative therapeutic approaches have been proposed such as progressive resistance training, dynamic balance training with proprioceptive exercises, mechanical stimulations, digital balance platforms, and treadmills [23–27]. On the other hand, aerobic training might be useful to enhance functional ability, aerobic capacity, strength, and fatigue in people with CMT [27–29]. Moreover, physiotherapy is important to maintain joint mobility, while the use of orthosis can enhance walking efficiency and reduce risk of falls [30–32].

Albeit rehabilitation is considered as a keystone for the treatment of the disease, the optimal exercise regime for people with CMT is not fully understood, and there is a lack of uniform guidelines [22, 33–35]. Furthermore, after rehabilitative intervention, home-exercise physical training is advised (about two and a half hours of aerobic exercise or activity per week, in 10-min bursts spread throughout the week) [22]. On other hand, the timing and the cadence of rehabilitative intervention are not clearly defined [26].

Recently, Ferraro et al. showed that intensive rehabilitation might improve functionality in patients affected by CMT1A treated with tailored functional surgery and lower limb cast, although the long-term effects were not investigated [36].

In this scenario, the aim of this retrospective study was to investigate effects of a 3-week intensive neurorehabilitation in terms of muscle strength and functioning in patients affected by CMT disease.

## Materials and methods

### Study design and setting

A retrospective analysis of patients’ records which participated in a rehabilitation program from September 2014 to June 2020 referring at the Presidio Riabilitativo Multifunzionale “Don Primo Mazzolari” (Bozzolo, Italy), a National referral center for CMT disease and for the Italian Charcot–Marie-Tooth Patient Association (ACMT-Rete).

The study was approved by the Val Padana Ethics Committee (registration number: 36-2021-OSS_ALTRO-MN13). All participants were asked to carefully read and sign an informed consent, and researchers provided to protect the privacy and the study procedures according to the Declaration of Helsinki, with pertinent National and International regulatory requirements. Moreover, the study was performed in accordance with the STrengthening the Reporting of OBservational studies in Epidemiology (STROBE) Guidelines [37].

### Participants

All the participants were patients affected by CMT who underwent rehabilitative treatment. Patients were diagnosed CMT by neurologist specialized in CMT before the rehabilitation program and outside the clinic. As patients were diagnosed outside the clinic, the criteria used for these diagnoses were not available.

Inclusion criteria were (1) clinical or genetic diagnosis of CMT; (2) ≥18 years old; (3) WHS score ≥3 (4) ability to understand and sign the informed consent form; (5) fully available da-ta for the duration of the study; (6) CMTNS scores ≤20 at baseline; and (7 ) respond to criteria for inpatients’ neuromuscular rehabilitation [38].

Exclusion criteria were (1) cognitive deficits or other psychological disorders that could affect the physiotherapy program or the physical evaluation (Mini-Mental State Examination ≤ 24); (2) limb surgery in the 12 months prior to treatment or screening; and (3) history of fractures in the previous 12 months.

### Data collection and measurements

We collected the following data for each patient: (1) age, (2) gender, (3) CMT type (demyelinating-axonal-other or mixed forms), (4) time from diagnosis, (5) Charcot–Marie-Tooth disease neuropathy score (CMTNS), (6) walking handicap scale (WHS), (7) Oxford handicap scale (OHS). CMTNS is a reliable and valid tool composite of nine assessments: 5 of impairment (“sensory symptoms,” “pin sensibility,” “vibration,” “strength arms,” and “strength legs”), 2 of activity limitations (“motor symptoms arms” and “motor symptoms legs”) and 2 electrophysiological measures (“ulnar CMAP” and “ulnar SNAP”). It is tailored to measure length-dependent motor and sensory impairment in genetic neuropathies. Each assessment is scored on a scale of 0–4 points, reflecting the severity of impairment. Patients are classified as mild (CMTNS ≤ 10), moderate (CMTNS 11–20), or severe (CMTNS > 20) [39]. The WHS is an evaluation tool that allows us to evaluate the walking handicap and disability in a domestic and social environment through a scale comprising six categories from minimum 1 and maximum 6.

All subjects provided demographic data. Clinical characteristics and type of CMT were obtained from medical records. The assessment involved a physiatrist and a trained physiotherapist (PT) that completed a clinical evaluation inclusive of the patient’s medical history, a physical examination, and objective and subjective measure scales, to identify a tailored rehabilitation program to be followed at our center.

### Outcome measures

The primary outcome of our study was the muscle strength, assessed in 6 muscles on either side for proximal lower limb muscles (ileo-psoas, quadriceps femoris, biceps femoris, gluteus maximus, medius, minimus) and for distal lower limb muscles (tibialis anterior, extensor digitorum communis, extensor hallucis longus, triceps surae, flexor hallucis longus, peroneus) using the MRC sum score (grades 0–60), considering as cut-offs: <48 for moderate or <36 for severe weakness [40, 41].

### Secondary outcomes were as follows

- Severity of pain, evaluated by a verbal rating scale (VRS), grading from 0 (minimum) to 10 (maximum);
- Severity of fatigue, evaluated by VRS, grading from 0 (minimum) to 10 (maximum);
- Severity of muscle cramps, evaluated by VRS, grading from 0 (minimum) to 10 (maximum);
- Balance ability, assessed through Berg Balance Scale (BBS), a 14-item objective test used to detect balance impairment and risk of fall in people affected by neuromuscular disorders, ranging from 0–56, where: 0, patient cannot stand alone, 0–44 risk of fall; 56, optimal stability [42];
- Walking ability and speed, evaluated by Walk12-scale, a patient-reported rating scale for walking difficulties in daily life, ranging from 0 (no difficulties during ambulation) to 60 (the worst troubles during walking) [43],
- Physical performance, evaluate by 10-m walking test (10 MWT), a validated test in CMT disease assessing functional mobility and walking speed in meters per seconds over a short duration in a set distance [44].
- At the baseline (T_0_), at the end of treatment (T_1_), and at 12 months (T_2_) all study participants were assessed for the following outcomes.

### Intervention

Physiotherapy program was administered for 3 weeks, 5 days a week, from 2 to 4 h a day according to patient’s fatigue. The protocol comprehended guided exercise, aerobic training, instrumental therapies, in particular electrical stimulation of affected muscles in all patients, and other analgesic physical therapies if needed, and patient self-care education. The intensive rehabilitation protocol is showed in Table 1.

**Table 1:** Rehabilitative protocol designed for complex intensive rehabilitation delivered to Charcot–Marie-Tooth patients

Treatment	Daily duration	Specific activities included in the program
PT-guided exercises	Two sessions per day, 1 h/session	**Passive manual mobilization** Mobility of foot joints, lower limb, and back
		**Stimulation of leg muscles** Leg muscles stimulated with manual facilitations according to the Bobath Concept, PNF, use of balance boards and balance exercises
		**Full-body stretching** Particular attention to the Achille’s tendon
		**Balance** Walking, training, and exercises in bipodal or monopodal stance, with the employment of balance boards and digital devices, head and eye movements, and double task exercises in different body positions
		**Core stability** Trunk-specific exercises
		**Resistance** Thigh and pelvic girdle muscles exercised in open and closed kinetic chains with ankle weights, elastic bands, or the subject’s own weight as a resistance
Self-administered activity	Daily, 10–60 min/session	**Aerobic activity** Exercise bike or treadmill training with a moderate intensity
Instrumental therapies	When necessary, 20–60 min/session	**Electrical stimulation** Applied to denervated lower limb muscles
		**Others** Lymphatic drainage, pressure therapy, magnetotherapy, laser therapy, and ultrasound therapy; all applied with analgesic aims
Patient education	During sessions and at the end of the rehabilitation period, 30 min	**Giving instructions and education about self-care to the patient** Drawings, videos, or pictures of specific exercises tailored to patient’s abilities and needs and suggestions about lifestyle and daily activity regime maintenance

**Fig. 1:**
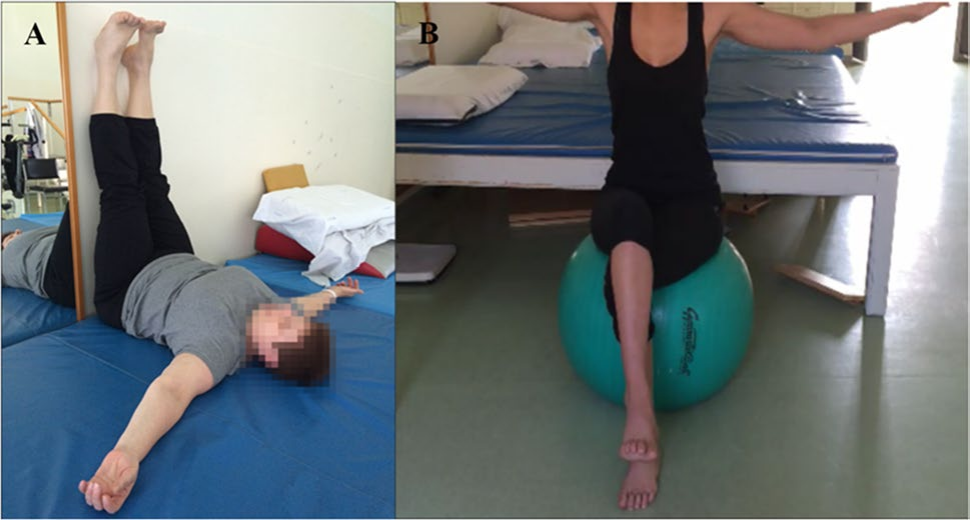
**a** Posterior chain muscle stretching that can be shortened in CMT patients. **b** Core stability exercise with ball in order to enhance stability

Each intervention was tailored to the patient’s needs and was adjusted daily to the patient’s symptoms and progress. During the treatments, great attention was paid to avoid overload weakness. An integral part of treatment was training patients on self-care activities aimed at maintaining beneficial effects of intensive physiotherapy program. The physiotherapist in charge of the intensive program gave each patient tailor-made exercises that included drawings, videos, and/or pictures. Moreover, advice on how to improve lifestyle and regular physical activity were given according to the patient’s abilities and needs (see Fig. 1).

The same team evaluated both the patient during the initial assessment and after the treatment program.

### Statistical analysis

Data analysis was performed using SPSS v.21.0 (SPSS Statistics, Armonk, NY: IBM Corp). Descriptive statistics was used to summarize data. Non-parametric tests were selected, based on a preliminary analysis of normality of data. The non-parametric Friedman’s ANOVA for repeated measures was used to analyze outcome value variations over time, followed by pairwise comparisons (T_0_ vs. T_1_, T_0_ vs. T_2_, and T_1_ vs. T_2_). Post hoc analysis with Wilcoxon signed-rank tests was conducted and was calculated the *R* effect size [45]. According to Cohen, the effect size is low if the value of *R* varies around 0.1, medium if *R* varies around 0.3, and large if *R* varies more than 0.5 [46]. Finally, a minimal clinical important difference (MCDI) of 4 was used for MRC sum scores [47].

## Results

Records by 37 CMT patients (14 males and 23 females) were analyzed (mean age of 50.72 ± 13.31 years). The mean time from diagnosis was 14.91 ± 14.08. Twenty-eight patients (75.68%) suffered from demyelinating CMT, 8 (21.62%) from axonal types of CMT, and 1 patient (2.70%) was affected by mixed form. The median score of CMTNS was 12 (7), with a moderate grade of disability. WHS score ranged between 3 and 6 with a median value of 5 (2), meaningful for independent walking with some limitations. The sample median score of Oxford handicap scale was 2 (1), standing for minor handicap and some restrictions in everyday life (see Table 2 for further details).

**Table 2:** Baseline characteristics of the study cohort

Age (years)	50.72 ± 13.31; 21–73
Gender (M/F)	14/23 *(37,8/62,2%)*
Time from diagnosis (years)	14.91 ± 14.08; 2–48
CMT type	*Demyelinating (28; 76%)* CMT1A: 16; CMTX: 5; CMT1B: 3; CMT:4 *Axonal (8; 22%)* CMT2A: 5; CMT2S: 1; CMT2C: 1; Axonal CMT: 1 *Other or mixed forms* (1; 3%)
CMTNS	12 (6); 3–19
WHS	5 (2); 3–6
OHS	2 (1); 1–3

Values are expressed in mean ± SD, median (IQR), and minimum–maximum

Abbreviations: *M* male, *F* female, *CMT* Charcot–Marie-Tooth disease, *CMTNS* Charcot–Marie-Tooth disease neuropathy score, *WHS* walking handicap scale, *OHS* Oxford handicap scale

More in detail, when considering the difference between single assessing time, all patients showed an improvement with a large effect size between T_0_ and T_1_ in MRC lower leg (*R* = 0.68), MRC upper leg (*R* = 0.81), NRS pain (*R* = 0.60), fatigue (*R* = 0.60), cramps (*R* = 0.61), BBS (*R* = 0.70), walk-12 (*R* = 0.59), and 10 MWT (*R* = 0.61), and after 1 year, we observed a small effect size for all the scales except VRS cramps (*R* = 0.30). On the other hand, there was a significant worsening with a large effect size in walking ability and speed, measured by in MRC lower leg (*R* = 0.49), and walk-12 (*R* = 0.70) compared to T_1_ (see Tables 3 and 4 for further details). When considering a MCID of 4 in MRC sum score variation, the number of subjects with strengthened lower leg muscles was 12 (32,4%, 95%CI: 16–45%) and 16 (43.2%, 95%CI: 25–54%) in upper leg muscles. It is worth noting that even subjects with MRC sum score = 0 at the baseline assessment could show increased strength at the end of the intensive rehabilitation protocol. No patients complained of more intense cramps at the 1-month follow-up, as depicted by Table 3.

**Table 3:** Differences in outcome measures after the rehabilitation treatment

Variables	T_0_	T_1_	T_2_	*p*-value
MRC sum score Lower leg	23.03 ± 19.25; 0–54	25.68 ± 20.47; 0–56	23.54 ± 20.36; 0–58	< 0.01
MRC sum score Upper leg	46.70 ± 9.45; 23–59	50.16 ± 7.85; 30–60	48.70 ± 8.35;28–60	< 0.01
VRS-pain	3.58 ± 3.23; 0–9	1.88 ± 2.31; 0–8	3.19 ± 3.44; 0–8	< 0.01
VRS-fatigue	4.56 ± 3.00; 0–9	3.41 ± 2.39; 0–8	4.25 ± 3.42; 0–9	< 0.01
VRS-cramps	2.47 ± 3.18; 0–9	0.88 ± 1.62; 0–7	1.86 ± 3.05; 0–10	< 0.01
Berg balance scale	37.67 ± 13.83; 13–56	41.91 ± 12.31; 20–56	36.75 ± 12.23; 21–56	< 0.01
Walk-12	12.75 ± 5.25; 12–60	11.25 ± 3.95; 12–48	13.05 ± 3.54; 18–55	< 0.01
10 MWT (m/s)	0.85 ± 0.29; 0.33–1.43	0.95 ± 0.26; 0.361.67	0.79 ± 0.20; 0.42–1.25	< 0.01

Values are expressed in mean ± SD and minimum–maximum; non-parametric Friedman’s ANOVA for repeated measures

Abbreviations: *VRS* verbal rating scale, *10 MWT* 10-m walking test

**Table 4:** *R* Effect size analysis of the outcome measures between the different time points

Outcomes	T_1_–T_0_	T_2_–T_0_	T_2_–T_1_
MRC sum score Lower leg	*R* = 0.68 (*p* < 0.01)	*R* = 0.06 (*p* = 0.71)	*R* = 0.49 (*p* < 0.01)
MRC sum score Upper leg	*R* = 0.81 (*p* < 0.01)	*R* = 0.19 (*p* = 0.25)	*R* = 0.23 (*p* = 0.16)
VRS-pain	*R* = 0.60 (*p* < 0.01)	*R* = 0.19 (*p* = 0.25)	*R* = 0.35 (*p* = 0.03)
VRS-fatigue	*R* = 0.61 (*p* < 0.01)	*R* = 0.11 (*p* = 0.49)	*R* = 0.27 (*p* = 0.10)
VRS-cramps	*R* = 0.50 (*p* < 0.01)	*R* = 0.30 (*p* = 0.07)	*R* = 0.21 (*p* = 0.21)
Berg balance scale	*R* = 0.70 (*p* < 0.01)	*R* = 0.28 (*p* = 0.09)	*R* = 0.46 (*p* < 0.01)
Walk-12	*R* = 0.59 (*p* < 0.01)	*R* = 0.12 (*p* = 0.45)	*R* = 0.20 (*p* < 0.01)
10 MWT (m/s)	*R* = 0.61 (*p* < 0.01)	*R* = 0.11 (*p* = 0.10)	*R* = 0.27 (*p* < 0.01)

Values are expressed in r effect size (*p*-value). We performed as statistical analysis using the Wilcoxon signed-rank tests. Effect size is low if the value of *R* varies around 0.1, medium if *R* varies around 0.3, and large if *R* varies more than 0.5

Abbreviations: *MRC* Medical Research Council, *VRS* verbal rating scale, *10 MWT* 10-m walking test

## Discussion

The aim of our retrospective study was to assess the short- and long-term effects of an in-patient intensive rehabilitation protocol on muscle strength and functioning in patients with mild to moderate CMT disease. No adverse effects occurred during the treatment period, as rehabilitation protocol might be safe and beneficial to CMT patients. Furthermore, remarkable improvements were found in muscle strength for distal and proximal muscles of the lower limbs after rehabilitation program. More in detail, the MRC sum score of the distal lower limb muscles showed a good effect size over the time (*R* = 0.68). On the other, it was interesting to note that the MRC sum score of proximal lower limb muscles significantly changed over time (*R* = 0.81).

According to the patient’s disease, the distal muscles were more compromised than the proximal ones. At the first evaluation, 23 (62.2%) showed severe and 11 (29.79%) moderate weakness in lower leg; 5 (13.5%) showed severe and 9 (24.3%) moderate weakness in upper leg; At the end of the intensive rehabilitation program, 19 (51.3%) and 6 (16.2%) patients showed severe and moderate weakness in lower limb, respectively. Similar results were found in upper leg muscle, 2 (5.4%) patients showed severe weakness and 7 (18.9%) moderate weakness.

Taking into account the MCID of 4 in terms of variation of MRC sum score, the 32.4% of CMT patients had strengthened proximal lower limb muscles and the 43.2% in distal lower limb leg muscles. These findings are in line with recent literature that supports the use of resistance training in CMT patients to increase muscle strength, whose levels could be also correlated to higher irisin levels that might represent in the future a marker of muscle mass loss and muscle strength loss [48–50].

Similarly, beneficial effect of the treatment was found for pain (*R* = 0.60). Reasonably, this contributed to improving the patients’ quality of life, since pain in CMT patients represents an important issue that impacts everyday life [21].

The cramps and the fatigue perceived by people with CMT are a relevant topic which is highly disabling but often poorly investigated [51]. We found a significant effect both in VRS cramps (*R* = 0.50) and fatigue (*R* = 0.61) after a tailored rehabilitative program. The reason for such a result can be mainly explained by the multifaceted rehabilitative program that includes both passive techniques and active exercises integrated with autonomous aerobic training [52, 53].

It is interesting to note that the greatest effect of a tailored rehabilitation program in CMT patients was found for balance at BBS (*R* = 0.70), demonstrating the feasibility and benefits of a complex and tailored intervention on postural stability. In literature, an improvement in balance in CMT patients had already been observed, both after single specific interventions aimed to enhance postural stability and after more complex rehabilitation treatments [24, 26, 54]. Postural instability issues should always be adequately addressed since these represent a relevant problem in CMT patients [55]. Falls inevitably lead to clinical complications that could prematurely cause a decline in a patient’s physical ability, thus reducing social participation and quality of life [56]. Being aware of the importance of monitoring fall risk in fragile patients, our results have proven to be strongly in favor of adopting rehabilitation protocols in the CMT population to counteract disease progression and therefore increase postural stability.

Moreover, we found a significant effect size in terms of walking ability and physical performance, as measured by the walk-12 (*R* = 0.59) scale and 10 MWT (*R* = 0.61), respectively. We hypothesized that the improvement in both balance and walking ability is linked to the increase in strength gained at the end of program. Faster walking, after resistance training of the lower limb proximal muscles, has already been observed in previous studies [55]. These results highlight the importance of rehabilitation, since walking ability strongly affects the quality of life in CMT patients [36, 56–58].

Despite positive results in the short term, almost all outcome values returned to baseline levels when assessing patients at T_2_. The lack of continuity in physical activity after discharge could also be the cause of the long-term decline in walking function and speed, as these outcomes are closely related to strength and postural stability.

Concerning the previous scientific literature, a systematic review [21] previously assessed the effects of rehabilitation in CMT patients: both affirm that, although benefits appear to be gained from exercise in strength and function in some studies, most outcomes reported were not significant. Moreover, the optimal exercise modality and intensity for people with CMT, the clinical relevance of the changes observed, and the safety of exercise are still unclear [21]. In 2020, Mori et al. [26] investigated the effect of treadmill along with stretching and proprioceptive exercises on balance and walking of CMT patients, with positive results at 3 and 6 months. Lastly, a case series shows that functional surgery integrated with early intensive neurorehabilitation might improve the gait performances of patients with CMT [36].

Therefore, it should be noted that the possible failure to maintain results in our cohort at the 1-year follow up. The possible failure to maintain results at 1 year follow up might show the need of enrolling in periodic intensive inpatient rehabilitation programs in specialized facilities. At the light of our results, an intensive rehabilitation program might play a central role in terms of reducing the disability in CMT and improving the functional status. In this context, our results suggested the need for an on-going long-term rehabilitation regime, which should not be interrupted at the end of the rehabilitation program.

In this scenario, the telerehabilitation might be an adequate and a more cost-efficient approach, considering its improvement during the COVID-19 pandemic and for the rehabilitation of other neurological diseases [58–61]. To the best of our knowledge, this is the first study to investigate the effects of a tailored rehabilitation protocol composed of mobilization, stretching, balance, and resistance exercises, with treadmill utilization and physical therapies, as electrical stimulation on lower limb muscles and other analgesic therapies at one year follow up, in a real practice approach, in CMT patient.

On other hand, we are aware that this study presents some limitations. First, the potential selection bias due to the retrospective study design and considering that only patients who had undergone all evaluation procedures and whose data were fully available were included. Second, the lack of a control group might be considered as a main limitation, albeit a control group might be in contrast with ethical reasons, whereby treatment must be guaranteed to all those who are deemed appropriate. In the future, we will provide an observational cross-over study. Third, the outcome measures are susceptible to several bias, such as little extra clinical information and remarkable floor/ceiling effect, creating a possible confounding effect in results. Fourth, we had no information regarding the adherence to the self-treatment or any activities that patients could have assumed after discharge in the period between T_1_ and T_2_. Lastly, patients’ lifestyles and other additional interventions not carried out at our facility have not been investigated.

## Conclusions

Taken together, our findings showed that a 3-week intensive rehabilitation treatment is a well-tolerated and useful intervention that might improve muscle strength and functioning in a cohort of inpatients with diagnosis of mild to moderate CMT. In this context, a continue exercise program seems to be necessary to avoid the functional loss. Specific rehabilitation strategies, such as increasing treatment frequency and supporting the patient’s self-care, are needed to sustain the improvements also in the long term in CMT patients.

## Data Availability

The data will be available with a reasonable request.
